# Supplementary material for: Cytotoxicity of Metal Ions Released from NiTi and Stainless Steel Orthodontic Appliances, Part 1: Surface Morphology and Ion Release Variations
Source: Materials (Basel). 2023 Jun 2;16(11):4156. doi: 10.3390/ma16114156 (PMC10254279; doi:10.3390/ma16114156)
Supplement: Supplementary file 1 [file materials-16-04156-s001.zip › materials-2395937-supplementary.pdf]

Table S1. Results of multi-element analysis of prepared samples of eluates of orthodontic appliances using high-resolution mass spectrometry with inductively coupled plasma. Artificial saliva eluates of metal ions released from archwires, brackets, ligatures, bands during three different elution periods (3, 7 and 14 days) were used for the analysis. The content of metal ions in artificial saliva (AS), which was used for preparation of all eluates in these experiments, is also given. The values are given in µg/l, as mean ± SD for all metals with concentration greater than 0.6 µg/l.

Table S1a) shows the metal ions (iron, nickel, chromium, manganese and titanium) with the highest concentrations determined.

**a)**	**Fe**	**Ni**	**Cr**	**Mn**	**Ti**
**Artificial Saliva**	3.72 ± 0.25	1.02 ± 0.02	0.09 ± 0.02	1.34 ± 0.01	0.82 ± 0.06
**NiTi archwires 3 days**	5.74 ± 0.12	199.17 ± 3.60	7.88 ± 0.44	1.46 ± 0.05	31.97 ± 0.75
**NiTi archwires 7 days**	10.36 ± 0.45	270.84 ± 1.61	0.81 ± 0.03	7.22 ± 0.11	21.30 ± 0.28
**NiTi archwires 14 days**	11.30 ± 0.30	38.61 ± 0.67	4.84 ± 0.12	7.01 ± 0.19	7.02 ± 0.24
**SS brackets 3 days**	231.53 ± 7.43	70.35 ± 0.81	10.35 ± 0.11	27.91 ± 0.28	0.74 ± 0.02
**SS brackets 7 days**	271.65 ± 5.37	71.33 ± 0.22	9.90 ± 0.19	27.17 ± 0.47	0.93 ± 0.10
**SS brackets 14 days**	146.60 ± 7.47	64.30 ± 0.43	9.21 ± 0.22	31.58 ± 0.51	0.76 ± 0.08
**SS ligatures 3 days**	137.66 ± 3.12	15.76 ± 0.28	5.15 ± 0.12	22.45 ± 0.33	1.05 ± 0.07
**SS ligatures 7 days**	122.38 ± 3.69	14.16 ± 0.26	5.36 ± 0.19	29.11 ± 0.54	1.02 ± 0.05
**SS ligatures 14 days**	127.69 ± 1.27	15.26 ± 0.31	4.29 ± 0.14	31.41 ± 0.49	25.39 ± 0.21
**SS bands 3 days**	928.75 ± 4.57	864.23 ± 12.50	569.24 ± 13.90	100.37 ± 1.89	0.34 ± 0.10
**SS bands 7 days**	2387.04 ± 31.30	1295.15 ± 16.80	722.89 ± 8.44	157.30 ± 4.02	0.65 ± 0.02
**SS bands 14 days**	1143.04 ± 13.60	1456.82 ± 13.00	411.46 ± 8.15	177.16 ± 2.64	0.34 ± 0.07

Table S1b) shows the sum of all determined the metal ions (Li, Be, Rb, Mo, Cd, Sn, Cs, Tl, Pb, Bi, U, Al, Ca, Ti, V, Cr, Mn, Fe, Co, Ni, Cu, Zn, Sr, Sb, Ba, As, Se).

**b)**	**Σ total detected metals**
**Artificial Saliva**	323.07 ± 10.09
**NiTi archwires 3 days**	518.83 ± 16.90
**NiTi archwires 7 days**	771.59 ± 13.22
**NiTi archwires 14 days**	951.65 ± 16.28
**SS brackets 3 days**	632.35 ± 15.94
**SS brackets 7 days**	740.32 ± 12.58
**SS brackets 14 days**	984.86 ± 25.53
**SS ligatures 3 days**	502.25 ± 9.51
**SS ligatures 7 days**	617.92 ± 17.63
**SS ligatures 14 days**	726.37 ± 18.29
**SS bands 3 days**	3069.94 ± 37.23
**SS bands 7 days**	6032.05 ± 80.44
**SS bands 14 days**	3753.94 ± 48.46

In Table S1.c) all determined biogenic metal ions were listed.

c)	**Ca**	**Co**	**Cu**	**Zn**	**Mo**	**Se**
**Artificial Saliva**	256.89 ± 8.90	0.03 ± 0.00	1.28 ± 0.02	14.45 ± 0.32	0.72 ± 0.01	0.101
**NiTi archwires 3 days**	216.27 ± 10.50	0.03 ± 0.00	1.25 ± 0.01	7.33 ± 0.43	1.17 ± 0.02	<0.002
**NiTi archwires 7 days**	317.07 ± 8.04	0.56 ± 0.01	0.61 ± 0.01	26.33 ± 0.53	7.90 ± 0.11	<0.002
**NiTi archwires 14 days**	531.04 ± 8.29	0.06 ± 0.02	1.39 ± 0.03	42.89 ± 0.28	1.04 ± 0.03	0.312
**SS brackets 3 days**	207.83 ± 5.57	2.33 ± 0.04	11.37 ± 0.19	19.85 ± 0.76	7.39 ± 0.05	0.075
**SS brackets 7 days**	251.59 ± 3.63	2.44 ± 0.07	11.50 ± 0.12	14.96 ± 0.54	8.12 ± 0.14	0.178
**SS brackets 14 days**	573.74 ± 13.40	2.23 ± 0.08	12.19 ± 0.46	38.66 ± 1.75	6.87 ± 0.04	0.034
**SS ligatures 3 days**	260.00 ± 4.62	0.60 ± 0.01	2.75 ± 0.04	8.34 ± 0.10	3.17 ± 0.04	0.041
**SS ligatures 7 days**	295.78 ± 11.00	0.56 ± 0.01	5.19 ± 0.02	26.49 ± 0.65	3.10 ± 0.04	0.476
**SS ligatures 14 days**	346.63 ± 12.50	0.61 ± 0.02	4.02 ± 0.08	36.80 ± 1.35	3.03 ± 0.04	0.180
**SS bands 3 days**	484.42 ± 2.29	19.78 ± 0.20	29.62 ± 0.56	21.26 ± 0.24	8.46 ± 0.15	<0.002
**SS bands 7 days**	573.29 ± 12.30	28.96 ± 0.85	25.84 ± 0.23	40.73 ± 0.07	10.73 ± 0.09	<0.002
**SS bands 14 days**	359.29 ± 8.69	29.75 ± 0.51	27.30 ± 0.37	44.31 ± 0.38	11.80 ± 0.11	0.073

In Table S1.d) all determined heavy metal ions were listed.

d)	Pb	Al	Sb	Cd	Sn	As	U	Bi
**Artificial Saliva**	2.98 ± 0.05	4.16 ± 0.14	0.08 ± 0.03	0.61 ± 0.01	0.098	0.084	0.079	0.011
**NiTi archwires 3 days**	0.89 ± 0.01	8.23 ± 0.26	0.12 ± 0.06	0.67 ± 0.02	0.056	0.061	0.059	0.002
**NiTi archwires 7 days**	2.19 ± 0.01	23.09 ± 0.96	0.65 ± 0.12	0.75 ± 0.01	0.102	0.102	0.057	0.008
**NiTi archwires 14 days**	6.75 ± 0.02	215.20 ± 4.44	0.81 ± 0.33	0.70 ± 0.00	0.086	0.089	0.051	0.035
**SS brackets 3 days**	0.14 0.08	4.98 ± 0.34	0.15 ± 0.03	0.69 ± 0.02	<0.002	0.067	0.039	0.001
**SS brackets 7 days**	4.53 ± 0.08	24.33 ± 0.72	0.61 ± 0.09	0.73 ± 0.01	0.011	0.101	0.045	0.010
**SS brackets 14 days**	2.40 ± 0.01	15.08 ± 0.32	0.72 ± 0.16	0.70 ± 0.02	0.005	0.087	0.031	0.009
**SS ligatures 3 days**	0.86 ± 0.01	7.09 ± 0.36	0.13 ± 0.02	0.66 ± 0.01	0.045	0.060	0.065	0.001
**SS ligatures 7 days**	4.77 ± 0.02	27.69 ± 0.42	0.83 ± 0.12	0.72 ± 0.01	0.035	0.032	0.052	0.007
**SS ligatures 14 days**	4.92 ± 0.04	27.46 ± 0.85	0.66 ± 0.50	0.75 ± 0.02	0.022	0.098	0.039	0.023
**SS bands 3 days**	0.27 ± 0.00	3.43 ± 0.10	0.48 ± 0.02	0.72 ± 0.01	<0.002	0.261	0.006	0.003
**SS bands 7 days**	679.46 ± 4.64	24.40 ± 0.74	0.36 ± 0.08	0.77 ± 0.01	0.058	0.348	0.019	0.025
**SS bands 14 days**	2.58 ± 0.02	11.14 ± 0.28	0.61 ± 0.04	0.71 ± 0.01	0.023	0.397	0.012	0.004

In Table S1.e) all the other determined metal ions were listed.

e)	Rb	Sr	Ba	V	Li	Tl	Be	Cs
**Artificial Saliva**	27.96 ± 0.09	4.84 ± 0.05	0.95 ± 0.03	0.15 ± 0.01	0.56 ± 0.01	0.042	<0.002	0.005
**NiTi archwires 3 days**	29.05 ± 0.38	5.30 ± 0.19	1.18 ± 0.06	0.16 ± 0.01	0.63 ± 0.01	0.042	0.13	0.005
**NiTi archwires 7 days**	69.56 ± 0.77	5.82 ± 0.09	5.05 ± 0.12	0.18 ± 0.01	0.37 ± 0.01	0.586	0.10	0.013
**NiTi archwires 14 days**	66.79 ± 0.88	6.38 ± 0.30	7.75 ± 0.33	0.19 ± 0.00	0.70 ± 0.02	0.557	0.06	0.012
**SS brackets 3 days**	28.80 ± 0.15	5.27 ± 0.05	1.23 ± 0.03	0.46 ± 0.02	0.63 ± 0.01	0.044	0.13	0.011
**SS brackets 7 days**	31.32 ± 0.53	5.50 ± 0.18	2.09 ± 0.09	0.48 ± 0.02	0.64 ± 0.00	0.048	0.12	0.016
**SS brackets 14 days**	67.48 ± 0.17	6.07 ± 0.32	4.45 ± 0.16	0.43 ± 0.01	0.59 ± 0.02	0.560	0.08	0.009
**SS ligatures 3 days**	29.04 ± 0.25	5.11 ± 0.04	1.12 ± 0.02	0.31 ± 0.01	0.60 ± 0.00	0.042	0.14	0.005
**SS ligatures 7 days**	68.85 ± 0.39	5.65 ± 0.15	4.47 ± 0.12	0.32 ± 0.01	0.20 ± 0.01	0.572	0.11	0.010
**SS ligatures 14 days**	72.94 ± 0.26	6.60 ± 0.03	15.66 ± 0.50	0.32 ± 0.02	0.85 ± 0.03	0.606	0.10	0.017
**SS bands 3 days**	29.89 ± 0.50	5.79 ± 0.13	0.53 ± 0.02	1.09 ± 0.03	0.90 ± 0.03	0.042	0.06	0.013
**SS bands 7 days**	70.03 ± 0.46	6.56 ± 0.24	4.56 ± 0.08	1.61 ± 0.02	0.62 ± 0.02	0.569	0.05	0.016
**SS bands 14 days**	66.59 ± 0.49	5.50 ± 0.03	1.63 ± 0.04	1.68 ± 0.04	1.14 ± 0.02	0.527	0.04	0.017
